# The Impact of Transcranial Magnetic Stimulation on Symptoms of Attention Deficit Hyperactivity Disorder and Sleep Parameters: Preliminary Study

**DOI:** 10.3390/bioengineering13030337

**Published:** 2026-03-13

**Authors:** Renato Mendes dos Santos, Francisco Victor Costa Marinho, Gabryella Stefane Firmino de Moraes, Sabrina Nayara de Araújo Val, Anderson César Ramos da Luz Carvalho, Leonardo Peres de Souza, Raimundo Pereira Silva-Néto, Victor Hugo do Vale Bastos, Silmar Silva Teixeira

**Affiliations:** 1 Neuro-Innovation Technology & Brain Mapping Laboratory, Federal University of Delta do Parnaíba, Parnaíba 64202-020, Piauí, Brazil; renatomendes@ufpi.edu.br (R.M.d.S.); gaby0bb12@gmail.com (G.S.F.d.M.); sabrina-nayara@hotmail.com (S.N.d.A.V.); silmarteixeira@ufdpar.edu.br (S.S.T.); 2 Brain Mapping and Functionality Laboratory, Federal University of Delta do Parnaíba, Parnaíba 64202-020, Piauí, Brazil; victorhugobastos@ufdpar.edu.br; 3 Genetics and Molecular Biology Laboratory, Federal University of Delta do Parnaíba, Parnaíba 64202-020, Piauí, Brazil; 4 State Department of Education and School Sports—SEDUC, Av. Waldomiro Lustoza, n.º 250, Bairro Japiim II, Manaus 69076-830, Amazonas, Brazil; andersoncesar88@hotmail.com; 5 Laboratory of Histological Analysis and Preparation, Federal University of Delta do Parnaíba, Parnaíba 64202-020, Piauí, Brazil; leoperes@ufpi.edu.br; 6 Postgraduate Program in Biomedical Sciences, Federal University of Delta do Parnaíba, Parnaíba 64202-020, Piauí, Brazil; netoesperantina@terra.com.br

**Keywords:** transcranial magnetic stimulation, time perception, attention deficit hyperactivity disorder, sleep quality, actigraphy

## Abstract

Background: Individuals with attention deficit hyperactivity disorder (ADHD) can exhibit neurocognitive deficits, psychosocial alterations, and changes in sleep regulation. In this sense, non-invasive techniques such as transcranial magnetic stimulation (TMS) can be used to assess and treat people with neurocognitive disorders. Studies using neuromodulation to estimate the timing and regulation of sleep remain scarce, revealing a gap in its understanding and treatment. Aim: To analyze whether the application of 10 Hz TMS modifies time estimation and sleep quality in people with ADHD for characteristics of inattention and hyperactivity. Material and Methods: Twelve adult male participants with high scores on the assessment of ADHD scale underwent a 10 Hz TMS protocol and a visual stimulus time estimation task. Daily rhythmicity was assessed by actigraphy before and after the repetitive transcranial magnetic stimulation (rTMS) intervention. Sleep quality was evaluated using the Pittsburg Sleep Quality Index and the Morning and Evening Questionnaire. Results: The results showed that rTMS modulated the interpretation of the 9 s long interval (*p* = 0.028). For the ADHD-AD instrument, no statistically significant differences were observed (*p* > 0.05). In relation to actigraphic variables—sleep time, bedtime, latency, sleep efficiency, and wakefulness after sleep onset—there were no differences after excitatory neuromodulation (*p* > 0.05). Conclusions: The findings demonstrate that the application of 10 Hz TMS enhances performance in long intervals during the time estimation task for individuals with ADHD, but does not affect sleep quality.

## 1. Introduction

The abilities of timing and sleep regulation are crucial for physiology, from hormonal regulation and activities related to motor and cognitive actions [1,2,3], with the execution of these activities through time intervals ranging from milliseconds to hours of the day [4,5]. In this context, these capacities are regarded as fundamental for all living organisms in their adaptation to the environment [6,7].

Time perception and sleep regulation in people with attention deficit hyperactivity disorder (ADHD) and in people who have not been accurately diagnosed and have reached adulthood with cardinal symptoms of inattention and hyperactivity have attracted considerable interest from researchers [8,9]. Diagnosed and undiagnosed ADHD are neurodivergent behaviors associated with a multifactorial condition that impairs the development of cognitive, emotional, and social aspects, including timing, sleep quality, and circadian rhythm [3,10]. Therefore, inattention and hyperactivity are considered characteristic phenotypes of ADHD patients, and there is evidence that these symptoms are partly caused by a hypo-functioning of the dopaminergic neurotransmission [11,12]. In this context, Scalar Expectancy Theory suggests that an accelerated internal clock may underlie abnormal time interval perception [13]. These studies have shown that neurodivergent disorders such ADHD are capable of inaccurately dissociating different durations of stimuli or anticipating motor actions after a specific time interval due to the reaction time being exacerbated by impulsivity [14,15,16,17,18]. In addition, sleep deprivation in adults with ADHD has shown an increase in daytime sleepiness, and a greater likelihood of showing a nocturnal circadian preference, and in this way, poor sleep quality is strongly associated with a variety of neurocognitive deficits and psychosocial impairments, resulting in difficulties in timing visual and auditory stimuli [19,20].

Several lines of rehabilitation and treatment can improve the cognition of individuals with ADHD, such as short-term treatment with psychostimulant drugs; however, they have side effects and limited long-term efficacy. Science is seeking non-invasive treatment methods that are more effective. Thus, neuromodulation by repetitive transcranial magnetic stimulation (rTMS) has proven to be a partial solution, showing promising results in improving cognitive functions in ADHD and subsequently aiding in improving sleep parameters [21,22,23].

rTMS represents a non-invasive treatment modality that operates by generating a magnetic field through brief, high-intensity electrical currents within a coil. This magnetic field penetrates the scalp and skull without attenuation, producing localized induced electric fields in target brain regions [24,25,26,27]. Several studies have demonstrated the effectiveness of transcranial magnetic stimulation in modulating cortical excitability [28]. High-frequency rTMS increases local neural excitability, and these excitatory effects enhance the brain’s inherent plasticity, allowing therapeutic applications in various neuropsychiatric conditions and association with cognitive task protocols [27,29,30].

The application of 10 Hz excitatory rTMS to the dorsolateral prefrontal cortex (DLPFC), which anatomically corresponds to Brodmann areas 9 and 46, stands out as the main target due to its relationship with cognitive processes involving time estimation through visual stimuli, and the treatment of psychiatric disorders and sleep disorders [26,27,30,31,32]. The state of the art has shown that excitatory brain stimulation of the DLPFC using rTMS can lead to improvements in emotional regulation [33]. This finding shows that emotional regulation can be effective and consequently acts on valence during cognitive control, and thus strengthens the argument in favor of improving cognitive abilities such as attention, learning, episodic memory, working memory, and both neurobiological aspects embedded in the perception of time [34,35]. There is evidence that neurostimulation in the DLPFC may play a specific role in the synaptic reinforcement of neural networks associated with sleep regulation, decision-making related to reward value and time perception, and, in particular, the inhibitory control of impulsive decision-making [36]. This cortical area exhibits hypoactivity in individuals with inattention and hyperactivity, and it is known that the DLPFC corresponds to an integrating center of executive functions, specifically involved in time estimation [35]. Therefore, considering the importance of the DLPFC in cognitive regulation and executive functions, brain stimulation at 10 Hz was performed in this region.

Although new research indicates that rTMS can have cognitive enhancement and sleep quality effects, the full scope of these effects and the factors that influence them remain unclear. To fill this knowledge gap, we conducted an experimental study to investigate whether rTMS modifies the pattern of time perception, the quality of sleep parameters and the circadian rhythm in people with ADHD.

## 2. Material and Methods

### 2.1. Participants

Twelve male participants were selected, with an average age of 23.4 ± 2.7 years. The study participants were characterized for ADHD in terms of inattention (score: 94.5 ± 5.3) and hyperactivity (score: 87.5 ± 13.2), as measured by the Attention Deficit Hyperactivity Disorder Scale, Adolescent and Adult Version (ADHDS-AD). All were right-handed according to the Edinburgh Handedness Inventory [37].

All participants underwent a medical assessment to exclude those with other neurological or motor diseases, visual, hearing, and motor impairments that would impair task performance, or who used any psychoactive or psychotropic substance during the study period. In addition, they completed the screening questionnaire for rTMS to identify conditions that could represent a risk factor for adverse effects. They were excluded if they had one or more positive responses in the screening [38].

### 2.2. Experimental Procedure

**Figure 1:**
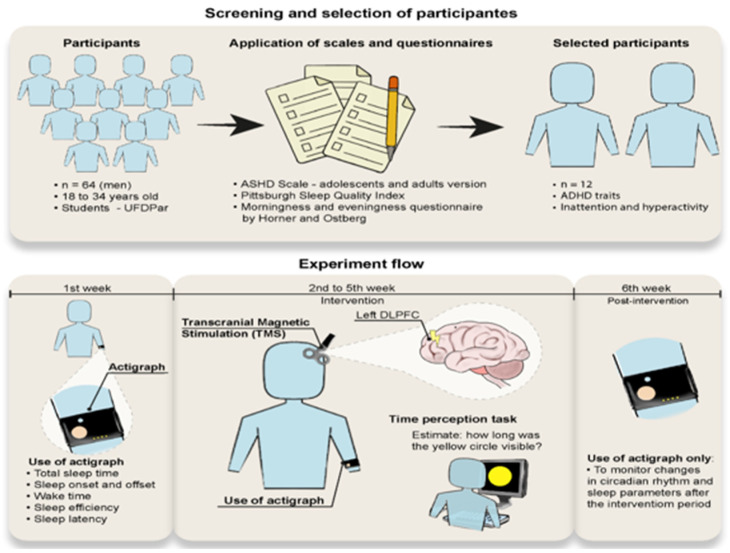
Study design: screening, selection of participants, and experimental protocol procedure.

**Figure 2:**
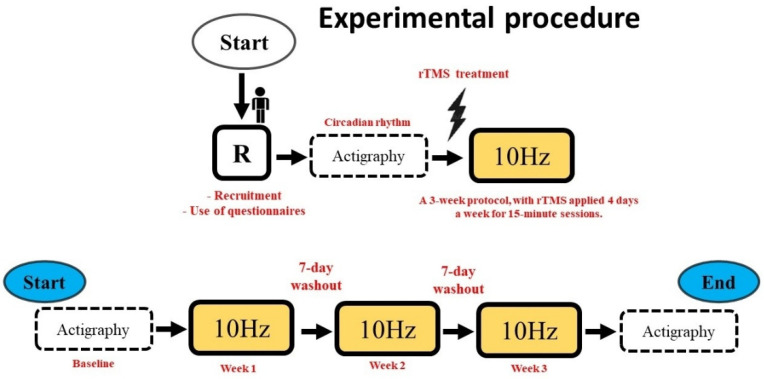
Experimental procedure.

This study had a cross-sectional design, with a treatment period of 4 days (rTMS stimulation at 10 Hz). Firstly, the subjects were selected based on the eligibility criteria according to the ADHDS-AD questionnaire. Subsequently, the sleep quality profile was assessed using the Pittsburg Sleep Quality Index (PSQI) and the Horner and Ostberg Morning and Evening Questionnaire. After applying the scales, actigraphy was collected for seven days to obtain the circadian rhythm baseline (Figure 1 and Figure 2).

Protocol: After collecting the actigraphic baseline, the experiment consisted of rTMS stimulation at 10 Hz for fifteen minutes in a room with acoustic and electrical insulation. A time estimation task was performed before and after rTMS stimulation. The protocol lasted three weeks, with brain stimulation occurring four days a week at a fixed time of 9:00 a.m. In addition, a seven-day wash-out phase was determined between each week of treatment. After the end of the protocol, actigraphy was collected for seven days in order to establish whether there had been any changes in the participant’s circadian rhythm (Figure 1 and Figure 2).

ADHDS-AD is made up of six factors: (a) inattention: related to attention skills, persistence, organization and rhythm in the performance of tasks; (b) impulsivity: related to deficits in the inhibitory system and low self-control; (c) emotional aspects: assesses the presence of emotional difficulties, related to a depressed mood, feeling of failure, among others; (d) self-regulation of attention, motivation and action: ability to maintain organization and planning; and (e) hyperactivity: refers to agitated, flustered and unstable behavior. The instrument helps in the process of identifying individuals with ADHD, with the possibility of distinguishing the presentation of the disorder, the intensity, and the level of impairment (mild: 30 to 39 points; moderate: 40 to 49 points; and higher: 50 points or more) [39].

### 2.3. Pittsburgh Sleep Quality Index Questionnaire (PSQI)

PSQI is considered the most applicable way of assessing sleep quality, as it is a short, accessible questionnaire that indicates people with good, bad, or disturbed sleep. The questionnaire assesses seven characteristic components of sleep quality, such as habitual sleep efficiency, sleep duration, sleep latency, sleep disturbances, use of sleep medication, daytime dysfunction, and qualitative aspects such as sleep activity, i.e., sleep duration and sleep repair capacity. Each component is assessed with a score ranging from 0 to 3. The total PSQI score is obtained by adding up the scores of the seven components, resulting in a total score ranging from 0 to 21, where scores above 5 indicate poor sleep quality [40].

### 2.4. Morning and Evening Questionnaire (MEQ)

To identify chronotypes, the Portuguese version of the MEQ was used, which was translated and adapted by the Multidisciplinary Group of Development and Biological Rhythms (MGDBR) of the Institute of Biomedical Sciences of the University of São Paulo [41]. The questionnaire consists of questions related to sleep habits and others related to people’s preferences for performing specific tasks during the day. Most of the questions in the questionnaire have four answer options. Some questions are asked using a set time interval. Each answer is given a score, indicating a low score for the afternoon type and a high score for the morning type. The final score, obtained after adding up the individual answers, generates a score that is categorized into five groups: extreme morning (70–86), moderately morning (59–69), intermediate (42–58), moderately afternoon (31–41) and extreme afternoon (16–30) [42].

### 2.5. Actigraphy

The test was conducted using a clock-like device (actigraph–Actrust 2-Condor Instruments, Brazil), which detects upper limb movements using an accelerometer system. As the method does not interfere with motor movement, it is easy to apply [43,44]. The parameters estimated are: total sleep time, sleep onset and end, wake time after sleep onset, sleep efficiency, and sleep latency.

The device used was the ActTrust Condor Instruments actimeter (Condor Instruments, São Paulo, Brazil), which monitors full-time exposure to light, temperature, and activity. ActTrust is equipped with a 3-axis accelerometer, featuring 12-bit resolution and a 25 Hz sampling rate. It includes sensors for accelerometer, wrist, and ambient temperature, as well as RGB-IR light. The configurable sampling interval ranges from 1 s to 86,400 s.

The actigraph sampling rate ranged from 0.5 to 10 Hz, epoch length: 30–60 s, and continuous recording: 7 days. This sampling range is sufficient to detect gross motor activity patterns relevant for sleep–wake classification while reducing battery consumption and data storage requirements.

### 2.6. Time Estimation Task

**Figure 3:**
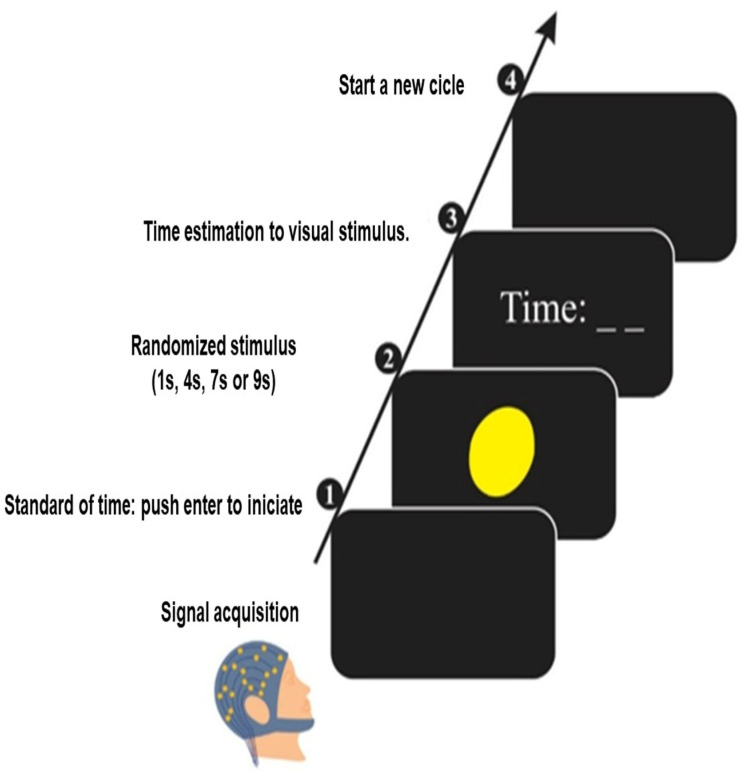
Experimental procedure for the time estimation task.

A 42-inch monitor (Philips Healthcare, Eindhoven, The Netherlands) was placed in front of the subjects at a distance of 70 cm and was turned on only during the task. We analyzed time estimation by visual stimulus with a program that records the displayed time interval (i.e., 1 s, 4 s, 7 s, or 9 s) [45]. We performed the task in two phases. In the first phase, the display shows the “enter” command to start. Then, the program produces a yellow circle in the center of the monitor, which is randomly displayed for 1 s, 4 s, 7 s, or 9 s. In the second, the software displays an empty field on the monitor to enter the estimated time interval, and then the subject presses the “enter” key to complete the task (Figure 3). Each subject performed 1 task block with 40 attempts.

### 2.7. Behavioral Data on Time Estimation

The behavioral variable relating the data with the onset of the visual stimulus to the end of the time estimation was transformed into measures representing the absolute error (AE) value and the estimated proportion to target duration (ratio). The AE value is defined as the difference between the target duration and the subjective time estimate [46]. Thus, the AE is a measure of the difference between the objective and judged clock pace, making it useful for assessing the overall level of accuracy of timing decisions [45,46,47,48]. The calculation is based on subtracting the target duration (Td) from each participant’s time performance (Tp), [AE = Td − Tp].

In addition, the ratio of the target duration estimate will be calculated by dividing each participant’s performance time by the duration of the target time interval presented for each track. This analysis corresponds to the ratio, which promotes the understanding that a coefficient below 1.0 indicates a judgment of time production that is shorter than the actual time. In contrast, a coefficient above 1.0 represents a judgment of time that is longer than the actual duration, i.e., an underestimation or overestimation of time, respectively [46]. The calculation is based on each participant’s time performance (Tp) divided by the target duration (Td) [Ratio = Tp/Td] [45,46,47].

### 2.8. rTMS Protocol

The rTMS pulses were emitted through a figure-of-eight coil with a diameter of 70 mm connected to a Neuro-Ms stimulator (medical equipment manufactured by Neurosoft, Ivanovo, Russia). First, before the rTMS session, the Resting Motor Threshold (rMT) was defined for each subject as the lowest stimulus intensity that promoted Motor Evoked Potentials (MEPs) with a peak-to-peak amplitude of more than 50 μV [49]. To determine the amplitude required for the use of rTMS, the coil was directed with the cable back to 45°. The single rTMS pulse at around 40% of the stimulator intensity was initially applied to the motor cortex [50]. The coil was moved around this reference point, corresponding to approximately 5 cm to the left of the vertex (i.e., electrode C3 of the 10-20 EEG system), to locate what would stimulate the MEP in the abductor pollicis brevis muscle of the right thumb recorded by electromyography. The intensity of the stimulator was gradually increased to record at least 5 out of 10 consecutive MEPs. After finding the rMT for each subject, this measurement was used as a reference to calculate the intensity of stimulation with rTMS. In total, 80% of the rMT was applied (mean = 46.2, SD = 9.12), as this intensity has been used as a safety measure to prevent seizures and yet has neuromodulatory effects [51].

rTMS was applied to the left dorsolateral prefrontal cortex, which was localized using the F3 electrode correspondence in the International 10-20 System. The left dorsolateral prefrontal cortex was chosen due to its association with attentional focus and memory, both neurobiological aspects embedded in the perception of time [26,27]. The coil was stabilized and immobilized by a mechanical support, an articulated 3D arm. The orientation of the coil was along the rostrocaudal axis, with an angulation of 45 degrees. All subjects wore earplugs. We applied a series of 900 stimuli at a rate of 10 Hz lasting 15 min.

### 2.9. Statistical Analysis

The normality and homoscedasticity of the data were previously verified by the Levene and Shapiro–Wilk tests. Statistical assumptions were respected. The descriptive statistics (mean, standard deviation, and 95% confidence intervals) were demonstrated for analyzing the behavior of variables ADHDS-AD and the sleep quality profile, as assessed by the PSQI, along with percentage evaluations for the MEQ instrument data.

Paired *t*-tests were carried out to analyze the differences before and after the 10 Hz rTMS intervention in the five domains of ADHDS-AD and for the PSQI.

The differences in absolute error and ratio in each time interval (1 s, 4 s, 7 s and 9 s) were carried out using Gamma tests, inserted into two-way Generalized Linear Models (GLMs) [52], with the following day factor: day 1 vs. day 2 vs. day 3 vs. day 4; and the following moment factor: before 10 Hz TMS vs. after 10 Hz TMS. In the context, Gamma distribution uses continuous, positive data tending towards asymmetry that must be met for a statistical test to produce valid, accurate results. The probability of 5% for type I error was adopted in all analyses (*p* ˂ 0.05), with alpha-Bonferroni correction for the interaction analysis, adjusting the *p*-value to *p* ≤ 0.0125.

Paired *t*-tests were used to test the differences before 10 Hz TMS vs. after 10 Hz TMS in the actigraphic variables (time in bed, sleep time, sleep latency, sleep efficiency, WASO and awakening). The analyses were conducted in SPSS for Windows version 20.0 (SPSS Inc., Chicago, IL, USA).

## 3. Results

### 3.1. Behavioral Profile Questionnaires

**Figure 4:**
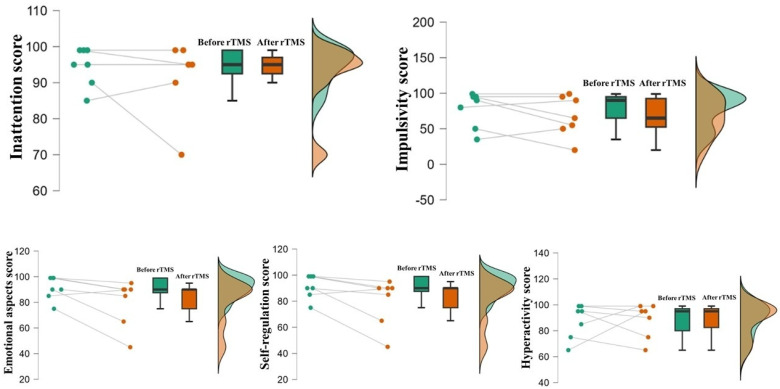
Raincloud plots of the ADHDS-AD instrument across the five domains. Individual data points connected by lines, boxplots, and density plots illustrate the distribution of scores before and after rTMS. Differences between time points were analyzed using the paired *t*-test. The plots show individual variability; however, no statistically significant differences were observed between pre- and post-rTMS assessments (*p* > 0.05).

**Figure 5:**
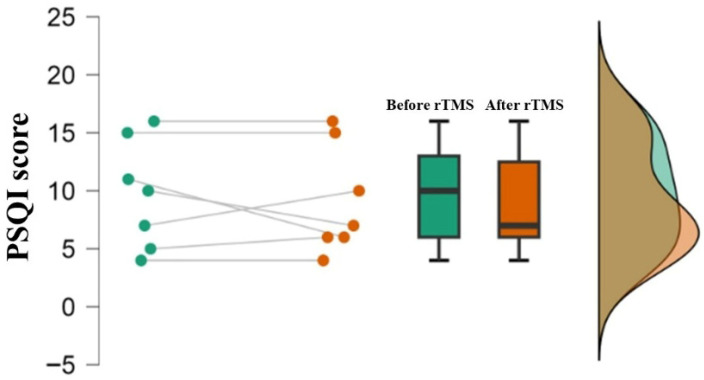
Raincloud plot of the PSQI score. Individual data points connected by lines, boxplots, and density plots illustrate the distribution of scores before and after rTMS. Differences between time points were analyzed using the paired *t*-test. The results show no statistically significant difference (*p* = 0.587).

The identification of ADHD characteristics using the ADHDS-AD instrument and the assessment of sleep quality using the PSQI before and after the 10 Hz TMS protocol were shown using descriptive analysis (Table 1, Figure 4 and Figure 5). In addition, the results of the MEQ instrument revealed that 76% of the sample had an intermediate chronotype profile. Subsequently, comparisons using the paired *t*-test for ADHDS-AD and PSQI showed no differences based on brain stimulation (*p* > 0.05). Although no statistically significant differences were observed after rTMS, descriptive measures suggested a reduction in scores for emotional aspects and impulsivity, as well as a modest change in self-regulation after the stimulation protocol.

### 3.2. Behavioral Variable of Time Perception

**Figure 6:**
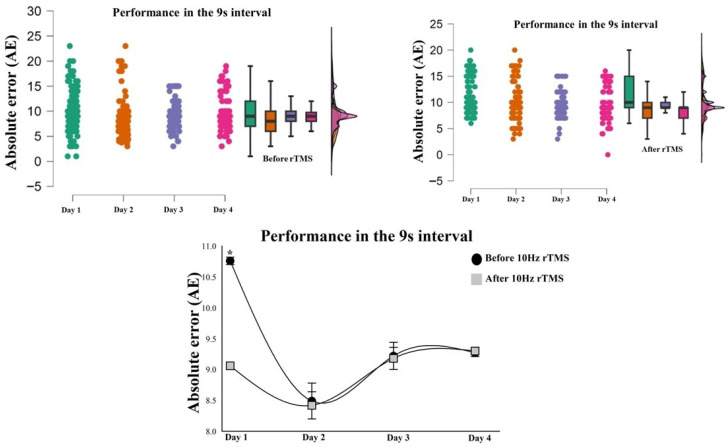
Raincloud plot of behavioral performance based on absolute error at the 9 s interval, before TMS and after 10 Hz rTMS. Individual data points connected by lines, boxplots, and density plots illustrate the distribution of scores across time points. Differences between day and moments were analyzed using a two-way Generalized Linear Model (GLM) with Gamma distribution. The results showed a statistically significant difference for the 9 s interval (*p* = 0.028). Note: * = The asterisk indicates a statistically significant difference.

The results show an increase in error as the time intervals increase, both before and after TMS on all days. The two-factor GLM Gamma tests for AE at 1 s, 4 s, and 7 s showed no interaction between the day vs. moment factor (*p* > 0.05). However, for the 9 s interval, the GLM Gamma test indicated an interaction, with χ^2^ = 9.06; *p* = 0.028, which shows differences between the moments for day 1 of the intervention, with an error of 1.7 s greater before brain stimulation (Figure 6). For the ratio, the Gamma GLM tests at intervals of 1 s, 4 s, 7 s, and 9 s showed no interaction between the day vs. moment factor (*p* > 0.05).

### 3.3. Actigraphic Variable

The sleep assessment measures are shown through the main descriptive measures in Table 2. In addition, the analysis of differences using the paired *t*-test before and after the TMS intervention protocol for time in bed, sleep time, sleep latency, sleep efficiency, wakefulness after sleep onset (WASO) and awakening did not show statistically significant results (*p* > 0.05): time in bed: [*t*(11) = 0.53, *p* = 0.625; 95%IC 0.031–0.059]; latency [*t*(11) = 0.06, *p* = 0.948; 95%IC 0.861 −0.914]; sleep efficiency [*t*(11) = 1.08, *p* = 0.301; 95% CI 1.441–4.272]; WASO [*t*(11) = 0.308, *p* = 0.764; 95% CI 0.212–0.282]; and WASO awakening [*t*(11) = 0.926, *p* = 0.347; 95% CI −3.21–1.32].

## 4. Discussion

This study analyzed the relationship between brain stimulation in the left dorsolateral prefrontal cortex and cognitive processes involving time estimation using 10 Hz rTMS in individuals with ADHD as a means of understanding the impacts on the interpretation of time estimation, sleep quality and circadian rhythm.

Characteristics such as inattention and hyperactivity are standard ADHD behaviors. In this context, the use of the ADHDS-AD instrument is an important means of psychological assessment, as it aims to identify the presence and intensity of ADHD characteristics [53], and with the joint action of the sleep quality and chronotype questionnaires, it increases the ability to understand cognitive, neurobiological and psychosocial aspects [19]. Although the ADHDS-AD domains did not show differences after rTMS, descriptive trends indicated reductions in scores related to emotional aspects and impulsivity, as well as changes in self-regulation. These patterns may suggest a potential modulation of emotional control and self-regulatory processes associated with dorsolateral prefrontal cortex stimulation. Since the DLPFC is involved in executive control and emotional regulation, these tendencies may reflect subtle neurocognitive modulations that could become more evident in studies with larger samples or controlled designs [53].

Regarding the interpretation of time intervals, the findings show that the rTMS protocol with a frequency of 10 Hz promoted modulation in the 9 s interval. This may be related to the greater cognitive demand for attention to the stimulus [45]. Consequently, the delays caused by inattention and hyperactivity in individuals with ADHD increase the speed of the internal clock, altering the neurotransmission of pulses and reactions to stimuli that determine synchronism in motor and cognitive activities [45,54,55]. Thus, individual variability in timing associated with brain stimulation can induce neurophysiological changes in perceptual mechanisms, since it modifies the proportion of neural activity required to interpret the time interval proposed by the task [56]. Thus, longer intervals that could be understood inaccurately can be adjusted to the perceptual average, as evidenced by the findings of this study. Based on this, it is understood that internal timers such as the left dorsolateral prefrontal cortex act as an information center, the processing of which depends on attention, since classifying the attentional reinforcement induced by stimulation at 10 Hz directs the focus to the relevant stimuli [57,58].

Suprasecond intervals can influence decision-making in people with inattention and hyperactivity [59,60]. Therefore, the timing basis for AE according to the Scalar Expectancy Theory demonstrates that the invariance of timescales attributed to the rate of pacemaker pulses and the transfer of temporal representations in memory may be deficient in individuals with ADHD, as it modulates the activity of cortical areas responsible for allowing the accumulation of information and reactions to stimuli, which are dependent on the level of neural arousal, emotional state and variations in the circadian cycle [61,62,63].

Thus, the low level of vigilance and attention before rTMS stimulation may indicate an inaccuracy in the time interval to be coded, and this implies a reduction in the speed of the internal clock, which influences cortical inputs and outputs in the synchronization of information relevant to perception [26,27]. This leads to temporal inconsistencies through low connective reinforcement between the prefrontal cortex and frontoparietal areas in processing information necessary for cognitive performance [6,64]. Thus, studies indicate that long intervals of time negatively affect decision-making capacity in individuals with ADHD, since difficulties in evaluating and responding to sequenced stimuli can lead to impulsive or inappropriate reactions [60]. Regarding decision-making, a study [65] showed that, when performing a task that requires more concentration and whose reward will not be immediate, there is less valence and motivational instability during the maintenance of the task, generating the desire to always look for something new [66]. These findings have important implications for the development of therapeutic protocols that consider the temporal structure of tasks and how rTMS maximizes benefits for people with ADHD [67]. Research into the effectiveness of rTMS on time perception indicates a specific heterogeneity, due to application in different cortical areas, changes in frequency, duration of application, and intensity of the stimulus [12,30].

Although the sleep pattern is not yet fully understood, the cognitive and behavioral changes resulting from sleep deprivation have been identified as factors associated with the cardinal features of ADHD. Alterations in noradrenergic and dopaminergic neurotransmitter pathways observed in individuals with ADHD, as well as metabolic modifications in the DLPFC, are also common in patients with sleep disorders [68]. The results for time in bed and sleep time indicate an inadequate number of hours, even though there were no differences after the intervention. This shows that people with ADHD have an increased sleep latency, a decrease in the percentage of REM sleep, and an increase in nocturnal motor activity. In this context, it is important to note that sleep is an essential state of consciousness for the regulation of energy metabolism and neurobiological functions, as it acts on memory consolidation and neural plasticity.

The findings show that, contrary to the recommended average sleep time of 8 to 9 h, individuals with characteristics of inattention and hyperactivity usually sleep an average of around 6.5 h, even after the application of rTMS. This reduction may include difficulties in initiating and maintaining sleep. Given this, adjustments to sleep habits are essential. Protocols that promote better sleep hygiene, such as consistent routines, reduced exposure to screens before bed and relaxation techniques, can offer significant benefits [69]. Although the results are not showing changes in sleep patterns, some studies have shown that the use of rTMS modifies sleep behavior patterns in people with ADHD [70,71]. The heterogeneity between findings can be attributed to the influence of individual factors, such as the severity of ADHD symptoms and the presence of comorbid sleep disorders.

The patterns observed in the actigraphy showed no changes in sleep behavior, focusing on sleep duration and continuity, time to fall asleep, time to wake up, and prediction of the individual’s chronotype. This indicates that, despite variations in the quality and quantity of sleep, changes in circadian rhythm may not be so marked in the short term, and the variability between individuals may mask the differences between the conditions analyzed [42,70]. While some may show obvious circadian dysregulations, others maintain sleep patterns that do not deviate significantly from what is expected, resulting in actigraphic data that do not reflect substantial changes [40,41,68].

### Study Limitations

The limited sample size, male-only sample, and the absence of both sham groups constrain statistical power and causal interpretation. Therefore, the observed effects cannot be unequivocally attributed to rTMS, as learning or expectancy effects may have contributed. Moreover, the effects were detected in only one time interval and assessment day, which limits the robustness and temporal stability of the findings.

This study did not include a sham rTMS condition, which represents an important methodological limitation. Although a sham stimulation would have strengthened causal inference by controlling for placebo and expectancy effects, the present design reflects a pragmatic clinical protocol. Future randomized sham-controlled trials are warranted to confirm the specificity of the observed effects.

Another limitation is the non-association with tasks at the sub-second level, since the Matlab—R2020b software used in the study does not provide for the possibility of stimuli with intervals below 1 s, as this could give a broader view of the behavioral performance associated with neuromodulation.

## 5. Conclusions

The present study is the first to analyze whether rTMS at 10 Hz modifies the estimation of time and sleep parameters by actigraphy in people with ADHD. The findings show that 10 Hz excitatory stimulation in the left DLPFC reduces the error in the timing of longer time intervals, adjusting accuracy during the perception of visual stimuli. However, rTMS did not alter the sleep quality of the study participants (maintaining a deficient sleep pattern) and ADHD symptoms.

Although the findings related to sleep patterns do not corroborate the research hypothesis, future studies with the rTMS protocol could act as a non-invasive clinical model for improving cognitive aspects and also as an adjuvant action in treatments already consolidated in the literature. In this way, it promotes the understanding of neurobiological aspects in people with ADHD, as well as in other neurological conditions.

## Figures and Tables

**Table 1:** Descriptive data from the ADHDS-AD instrument in the five domains and sleep quality using the PSQI before and after rTMS. Paired *t*-tests were carried out to analyze the differences before and after the 10 Hz rTMS intervention in the five domains of ADHDS-AD and for the PSQI. Note: SD: Standard deviation; 95% CI: 95% confidence interval; SEM: standard error of the mean; MD: mean difference.

**ADHD-AD instrument**							
**Before rTMS**		**After rTMS**					
Inattention		Inattention		Paired *t*-test			
Mean ± SD	95% CI	Mean ± SD	95% CI	MD ± SD	SEM	95% CI	*p*-value
94.5 ± 5.3	89.6–99.5	91.8 ± 10.1	82.5–101.2	2.7 ± 8.05	3.04	−4.73–10.2	0.407
Impulsivity		Impulsivity		Paired *t*-test			
Mean ± SD	95% CI	Mean ± SD	95% CI	MD ± SD	SEM	95% CI	*p*-value
77.7 ± 25.1	54.4–100.9	67.7 ± 28.8	41.7–94.3	10.0 ± 21.01	7.94	−9.43–29.43	0.255
Emotional aspects		Emotional aspects		Paired *t*-test			
Mean ± SD	95% CI	Mean ± SD	95% CI	MD ± SD	SEM	95% CI	*p*-value
91.0 ± 9.0	82.6–99.3	81.2 ± 19.3	63.3–99.1	9.8 ± 13.4	5.48	−2.75–25.42	0.094
Self-regulation		Self-regulation		Paired *t*-test			
Mean ± SD	95% CI	Mean ± SD	95% CI	MD ± SD	SEM	95% CI	*p*-value
49.2 ± 26.3	24.9–73.6	54.4 ± 22.4	33.6–75.2	5.2 ± 12.3	4.6	−0.36–22.4	0.056
Hyperactivity		Hyperactivity		Paired *t*-test			
Mean ± SD	95% CI	Mean ± SD	95% CI	MD ± SD	SEM	95% CI	*p*-value
87.5 ± 13.2	75.3–99.7	88.2 ± 13.2	76.1–100.4	0.7 ± 16.04	6.23	−15.97–14.15	0.913
**PSQI instrument**							
**Before rTMS**		**After rTMS**		Paired *t*-test			
Mean ± SD	95% CI	Mean ± SD	95% CI	MD ± SD	SEM	95% CI	*p*-value
9.71 ± 4.68	5.38–14.04	9.14 ± 4.7	4.79–13.49	0.57 ± 2.63	0.99	−1.86–3.01	0.587

**Table 2:** Description of actigraphic variables before and after the 10 Hz rTMS protocol. Paired *t*-tests were used to test the differences before 10 Hz TMS vs. after 10 Hz TMS in the actigraphic variables (time in bed, sleep time, sleep latency, sleep efficiency, WASO and awakening). Note: SD: Standard deviation; SEM: standard error of the mean; 95% CI: 95% confidence interval; MD: mean difference.

Before 10 Hz rTMS			After 10 Hz rTMS						
Time in bed			Time in bed			Paired *t*-test			
Mean ± SD	SEM	95% CI	Mean ± SD	SEM	95% CI	MD ± SD	SEM	95% CI	*p*-value
0.282 ± 0.06	0.19	0.240–0.324	0.291 ± 0.04	0.01	0.262–0.321	0.009 ± 0.06	0.04	−0.02–0.05	0.625
Sleep time			Sleep time			Paired *t*-test			
Mean ± SD	SEM	95% CI	Mean ± SD	SEM	95% CI	MD ± SD	SEM	95% CI	*p*-value
0.244 ± 0.06	0.01	0.208–0.281	0.251 ± 0.04	0.01	0.220–0.282	0.007 ± 0.05	0.02	−0.04–0.03	0.688
Efficiency			Efficiency			Paired *t*-test			
Mean ± SD	SEM	95% CI	Mean ± SD	SEM	95% CI	MD ± SD	SEM	95% CI	*p*-value
86.5 ± 7.91	2.82	81.5–91.5	85.1 ± 6.34	1.82	81.09–89.14	0.95 ± 4.49	1.29	−1.44–4.21	0.300
Latency			Latency			Paired *t*-test			
Mean ± SD	SEM	95% CI	Mean ± SD	SEM	95% CI	MD ± SD	SEM	95% CI	*p*-value
1.991 ± 1.12	0.32	1.275–2.707	2.02 ± 1.53	0.44	1.04–2.99	0.029 ± 1.39	0.40	−0.91–0.86	0.948
WASO			WASO			Paired *t*-test			
Mean ± SD	SEM	95% CI	Mean ± SD	SEM	95% CI	MD ± SD	SEM	95% CI	*p*-value
0.705 ± 0.641	0.18	0.297–1.127	0.739 ± 0.53	0.15	0.401–1.078	0.034 ± 0.38	0.11	−0.28–0.21	0.764
Awakening			Awakening			Paired *t*-test			
Mean ± SD	SEM	95% CI	Mean ± SD	SEM	95% CI	MD ± SD	SEM	95% CI	*p*-value
9.45 ± 6.37	1.83	5.44–13.53	10.44 ± 5.40	1.55	7.01–13.87	0.99 ± 3.56	1.03	−3.21–1.13	0.374

## Data Availability

The raw data supporting the conclusions of this article will be made available by the authors on request.
